# Chromosome-Scale Assembly of the Complete Genome Sequence of Leishmania (Mundinia) procaviensis Isolate 253, Strain LV425

**DOI:** 10.1128/mra.01306-22

**Published:** 2023-03-06

**Authors:** Hatim Almutairi, Michael D. Urbaniak, Michelle D. Bates, Godwin Kwakye-Nuako, Waleed S. Al-Salem, Rod J. Dillon, Paul A. Bates, Derek Gatherer

**Affiliations:** a Division of Biomedical and Life Sciences, Faculty of Health and Medicine, Lancaster University, Lancaster, United Kingdom; b Ministry of Health, Riyadh, Saudi Arabia; c Department of Biomedical Sciences, School of Allied Health Sciences, College of Health and Allied Sciences, University of Cape Coast, Cape Coast, Ghana; University of California, Riverside

## Abstract

Leishmania (Mundinia) procaviensis is a parasitic kinetoplastid that was first isolated from a rock hyrax in Namibia in 1975. We present the complete genome sequence of Leishmania (Mundinia) procaviensis isolate 253, strain LV425, sequenced using combined short- and long-read technologies. This genome will contribute to our understanding of hyraxes as a *Leishmania* reservoir.

## ANNOUNCEMENT

Leishmaniasis is a kinetoplastid parasitic disease with clinical manifestations ranging from localized cutaneous lesions to life-threatening, widespread, visceral infection (1–3). Rodents are an important group among several animal reservoir hosts (4), and *Leishmania* parasites have been isolated from hyraxes (family Procaviidae) in Ethiopia (5), Saudi Arabia (6), Israel (7), and Namibia (7). Here, we report the complete genome assembly and annotation for Leishmania (Mundinia) procaviensis (8) isolate 253, strain LV425 (WHO code MPRO/NA/1975/253;LV425), which was isolated from a rock hyrax (Procavia capensis) in Namibia in 1975 (9). This genome will contribute to our understanding of hyraxes as a reservoir for *Leishmania*.

Parasites were grown in Schneider's insect medium at 26°C as promastigotes and then in M199 medium (Sigma-Aldrich) supplemented with 10% fetal calf serum (FCS), 2% stable human urine, 1% basal medium Eagle vitamins, and 25 μg/mL gentamicin sulfate, with subpassage to fresh medium every 4 days to sustain parasite growth and viability (10). DNA was extracted and purified using a Qiagen DNeasy blood and tissue kit with the spin column protocol, according to the manufacturer’s instructions. The extracted DNA concentration was assessed using a Qubit fluorometer, microplate reader, and agarose gel electrophoresis. All sequencing libraries were based on the same extracted DNA sample to avoid any inconsistency.

Short-read library construction and sequencing were contracted to (i) BGI (Shenzhen, China) (paired-end reads [270 bp and 500 bp] sequenced using the Illumina HiSeq platform) and (ii) Aberystwyth University (Aberystwyth, UK) (paired-end reads [300 bp] sequenced using the Illumina MiSeq platform). We performed long-read library preparation and sequencing according to the Nanopore protocol (SQK-LSK109) on R9 flow cells (FLO-MIN106). Read quality was assessed using MultiQC (11).

We assembled the long reads with Flye (12), using default parameters, to generate chromosome-scale scaffolds. Then, using Minimap2 (13) and SAMtools (14), we mapped the short reads onto the assembled scaffolds to compensate for erroneous bases within the long reads and to create consensus sequences. After polishing of the assembly with Pilon (15), another round of consensus short-read mapping was performed. Then, we removed duplicate contigs and sorted the remainder of the contigs according to length using Funannotate (16). Finally, we separated the chimeric sequences and performed scaffolding using RaGOO (17) with the Leishmania major Friedlin strain genome (GenBank assembly accession number GCA_000002725.2) (18) as a reference guide, aligning all 36 chromosomes for our assembly with the exception of 31 unplaced contigs totaling 248,213 bp.

**FIG 1:**
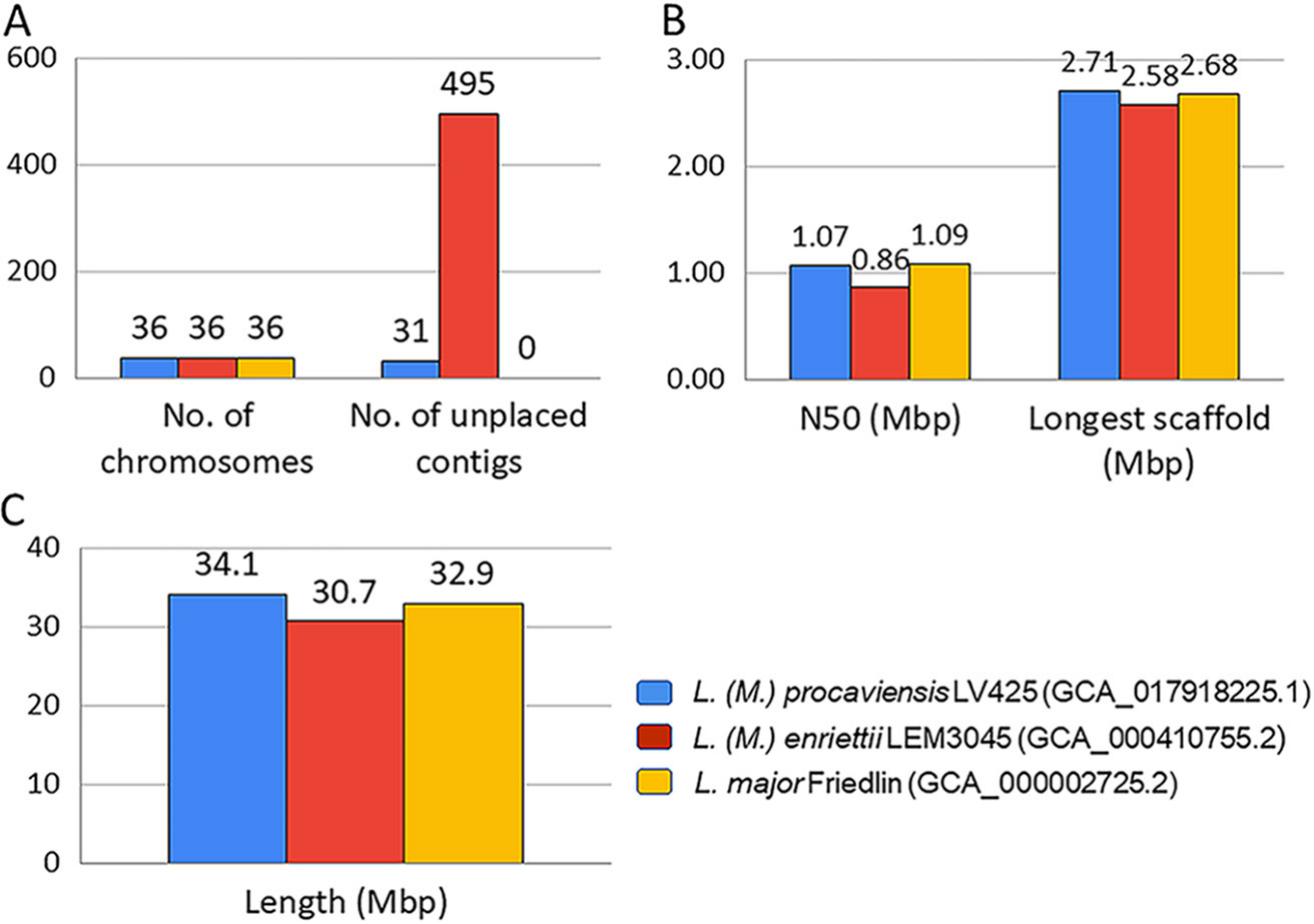
Assembly comparison of L. (M.) procaviensis LV425 with Leishmania (Mundinia) enriettii LEM304 and L. major Friedlin.

The analysis workflow for assembly and annotation was performed using Snakemake (19) and is available online for reproducibility purposes (20), including software versions and parameters used. Figure 1 compares our assembly with other complete genomes.

We assessed assembly completeness with BUSCO (21), using the lineage data set for phylum *Euglenozoa*, which contains 130 single-copy orthologues from 31 species; we found that 123 of the orthologues were present (94.6% completeness). We carried out functional annotation and prediction using the MAKER2 annotation pipeline (22) in combination with AUGUSTUS gene prediction software (23). Table 1 shows further summary metrics for sequencing, assembly, and annotation.

**TABLE 1:** Detailed summary of the genome sequencing, assembly, and annotation metrics for L. (M.) procaviensis LV425

Feature	Finding
Total no. of reads	29,347,348
No. of MiSeq reads	13,467,308
No. of HiSeq reads	15,113,450
No. of MinION reads (read *N*_50_ [bp])	766,590 (15,710)
Total read size (Gbp)	20.51
Genome coverage (×)	291.5
Total no. of scaffolds	67
Genome size (bp)	34,118,624
*N*_50_ (bp)	1,066,046
GC content (%)	59.50
No. of Ns (% of genome)	530 (0.002)
No. of genes	8,266
Gene density (genes/Mb)	243.2
No. of exons	8,529
Mean gene length (bp)	1,919
Total length of coding sequences (Mb [% of genome])	15.48 (45.37)

### Data availability.

The assembly and annotations are available under GenBank assembly accession number GCA_017918225.1. The master record for the whole-genome sequencing project is available under GenBank accession number JAFNID000000000.1. Raw sequencing reads are available under BioProject accession number PRJNA689706.
